# Supplementary material for: From Voxel to Gene: A Scoping Review on MRI Radiogenomics’ Artificial Intelligence Predictions in Adult Gliomas and Glioblastomas—The Promise of Virtual Biopsy?
Source: Biomedicines. 2024 Sep 23;12(9):2156. doi: 10.3390/biomedicines12092156 (PMC11429468; doi:10.3390/biomedicines12092156)
Supplement: Supplementary file 1 [file biomedicines-12-02156-s001.zip › biomedicines-3161074-supplementary.pdf]

biomedicines

MDPI

Supplemental material

From Voxel to Gene: a Scoping Reviews on MRI Radiogenomics Artificial Intelligence Predictions in Adult Gliomas et Glioblastomas - the Promise of Virtual Biopsy?

1. S1: Request Review

1.1 PubMed

(((Glioma[MeSH]) OR (Glioblastoma[MeSH]) OR (Brain Neoplasms[MeSH]) OR (Glioma[Title/Abstract]) OR (Glioblastoma[Title/Abstract]) OR (Gliomas[Title/Abstract]) OR (Glioblastomas[Title/Abstract]) OR ("Brain Tumor"[Title/Abstract]) OR ("Brain Tumors"[Title/Abstract]) OR ("Central Nervous System Tumors"[Title/Abstract]) OR ("CNS Tumors"[Title/Abstract]))

AND

(("radiogenomic"[Title/Abstract]) OR ("radiogenomics"[Title/Abstract]) OR ("imaging genomics"[Title/Abstract]) OR ("imaging genetics"[Title/Abstract]))) OR ((("radiogenomic"[Title/Abstract]) OR ("radiogenomics"[Title/Abstract]) OR ("imaging genomics"[Title/Abstract]) OR ("imaging genetics"[Title/Abstract]))

1.2 EmBase

('glioma'/exp OR 'brain tumor'/exp OR glioma:ti,ab,kw OR glioblastoma:ti,ab,kw OR gliomas:ti,ab,kw OR glioblastomas:ti,ab,kw OR 'brain tumor*':ti,ab,kw OR 'central nervous system tumor*':ti,ab,kw OR 'cns tumor*':ti,ab,kw OR 'central nervous system tumor'/exp)

AND

('radiogenomics'/exp OR 'imaging genetics'/exp OR 'radiogenomic':ti,ab,kw OR 'radiogenomics':ti,ab,kw OR 'imaging genomics':ti,ab,kw OR 'imaging genetics':ti,ab,kw)

1.3 Cochrane

#1 ("glioma"):ti,ab,kw OR ("glioblastoma"):ti,ab,kw OR ("brain tumor"):ti,ab,kw OR (Central Nervous System Tumors):ti,ab,kw OR (CNS Tumors):ti,ab,kw

#2 (radiogenomic):ti,ab,kw OR (radiogenomics):ti,ab,kw OR (imaging genomics):ti,ab,kw

#3 (#1 AND #2)
